# The effects of different restorative materials on periodontopathogens in combined restorative-periodontal treatment

**DOI:** 10.1590/1678-7757-2017-0154

**Published:** 2018-01-24

**Authors:** Sila Çagri ISLER, Gonen OZCAN, Gülcin AKCA, Zahide KOCABAS

**Affiliations:** 1 Gazi University, Faculty of Dentistry, Department of Periodontology, Ankara, Turkey.; 2 Gazi University, Faculty of Dentistry, Department of Medical Microbiology, Ankara, Turkey.; 3 Ankara University, Faculty of Agriculture, Biometry and Genetics Unit, Ankara, Turkey.

**Keywords:** Bacteria, Tooth abrasion, Connective tissue, Dental restoration, Biofilms

## Abstract

**Objective:**

The aim of the study was to evaluate the association between subgingival restorations and the target periodontopathogenic bacteria (Pg, Td and Pi) in subgingival biofilm during one year after combined restorative-periodontal treatment.

**Material and Methods:**

Seventeen systemically healthy subjects, who were positive for the presence of three cervical lesions associated with gingival recessions in three different adjacent teeth, were included in the study. A total of 51 combined defects were treated with connective tissue graft plus a nanofilled composite resin (NCR+CTG), a resin-modified glass ionemer cement (RMGI+CTG) and a fluoride-releasing resin material with pre-reacted glass (PRG), called giomer (Giomer+CTG). Periodontal clinical measurements and subgingival plaque samples were obtained from all combined defects at baseline and at 6 and 12 months after the surgery. The number of bacteria were evaluated by the real-time polymerase chain reaction (qPCR) method.

**Results:**

No statistically significant difference in the amount of DNA copies of Pg, Td and Pi was observed in any of the groups at any time points (p>0.05). In addition, there was no statistically significant difference in the amount of DNA copies of the bacteria at baseline and at 6 and 12 months postoperatively, regardless of treatment group (p>0.05).

**Conclusion:**

This study suggests that subgingivally placed NCR, RMGI and giomer restorations can show similar effects on periodontopathogenic bacteria in the treatment of gingival recessions that are associated with noncarious cervical lesions (NCCLs).

## Introduction

Gingival recessions and noncarious cervical lesions (NCCLs) are frequently associated with the same tooth surface, forming a combined defect, and are closely related^34^. These combined defects result in numerous aesthetic and functional problems, and a comprehensive treatment approach is required to address the issue. A combined restorative-periodontal therapy, in which the restorative therapy is completed before mucogingival surgery, has been proposed for the treatment of gingival recession that is associated with NCCLs^14^ ^,^ ^27^ ^,^ ^36^. Following the healing period after surgery, the soft tissue is positioned over a part of the restorative material and the apical border of the restoration is in the subgingival area. However, the response of the gingival tissues to the restorative materials is very important, and this relationship has been thoroughly investigated over many years^18^. It has been reported that subgingival restorations are associated with greater plaque accumulation, bleeding on probing, and attachment loss^17^, while other studies have indicated that the restorations do not result in greater biofilm formation, bacterial accumulation and clinical attachment loss, compared with non-restored areas^7^ ^,^ ^23^ ^,^ ^28^.

Bacterial composition on subgingival restorations can trigger the development of periodontal disease. It has been suggested that some members of this composition, known as “keystone pathogens”, could regulate biofilm virulence and modulate the host immune response^9^ ^,^ ^11^ ^,^ ^13^. Longitudinal studies have shown that periodontal disease progression can be predicted by the levels of *Porphyromonas gingivalis* (*Pg*) and *Treponema denticola* (*Td*) in subgingival plaque^3^ ^,^ ^9^ ^,^ ^12^. Moreover, it has been reported that *Pg* and *Prevotella intermedia* (*Pi*) are more frequently associated with deeper periodontal pockets^31^.

Various dental materials and surgical approaches have been used to manage these combined defects, in order to provide the most predictable combined restorative-periodontal treatment^14^. In this treatment method, resin composites or resin-modified glass ionomer cements (RMGIs) have been commonly used to restore NCCLs^19^, and gingival recessions have been treated using the coronally advanced flap (CAF) technique, either alone or in combination with a connective tissue graft (CTG)^14^ ^,^ ^21^ ^,^ ^24^ ^,^ ^28^. Some of the previous studies evaluated the effects of subgingivally placed restorative materials on periodontopathogenic bacteria in the combined restorative-periodontal treatment^23^ ^,^ ^28^. However, there is a lack of information in the current literature regarding the effect of subgingival restorations that are carried out using nanofilled composite resin (NCR), RMGI and giomer on periodontopathogenic bacteria in the treatment of gingival recessions associated with NCCLs.

The primary objectives of this study were to evaluate the association between subgingival NCR, RMGI and giomer restorations and three periodontopathogenic bacteria (*Pg*, *Td* and *Pi*) in subgingival biofilm during one year after combined restorative-periodontal treatment, and to examine the correlations between these pathogens and the clinical data.

## Material and methods

### Study design and population

This was a prospective, 12-month split-mouth clinical study. A total of 23 individuals, who were admitted to the Department of Periodontology, at the Faculty of Dentistry, Gazi University, Ankara, Turkey, were referred for treatment of gingival recessions associated with NCCLs. The study protocol was approved by the Ethics Committee of the Faculty of Medicine, Gazi University (Protocol ID: 25901600-7587). The participants were then informed of this protocol and their written informed consent was obtained.

The participants were included on the basis of the following inclusion criteria: (1) age >18 years, (2) positive for the presence of three cervical lesions associated with multiple gingival recessions in three different adjacent teeth, excluding molars, (3) Miller Class I gingival recession defects (≥2 and ≤5 mm) associated with buccal NCCL Class B + step^20^, (4) NCCL depth of 1–2 mm, (5) non-smoker, (6) systemically healthy (7) and probing depth (PD) ≤3 mm.

NCCLs were randomly allocated to three treatment groups using a computer-generated randomization scheme, as follows: NCR+CTG group, in which the combined defects were restored with NCR and treated by CTG; RMGI+CTG group, in which the combined defects were restored with RMGI and treated by CTG; Giomer+CTG, in which the combined defects were restored with giomer and treated by CTG.

The sample size was calculated considering a 0.5 log difference in counts of DNA copies of bacteria within the treatment groups, with estimated standard deviation of 0.5 in each group, an effect size of 0.4 and a power of 82.3%, using a factorial repeated-measures analysis of variance test. This would require 16 combined defects in each group.

### Clinical measurements

Periodontal clinical measurements and subgingival plaque samples were obtained by an examiner that was blinded to the treatment allocation. All of the following clinical measurements were recorded immediately before surgery (baseline), and at 6 and 12 months after surgery: (1) plaque index (PI)^16^; (2) bleeding on probing (BOP)^1^; (3) probing depth (PD) measured as the distance from the gingival margin to the bottom of the probe-able pocket; (3) relative recession height (rRH) measured as distance from the most apical point of gingival margin to the incisional border of the tooth; (4) relative clinical attachment level (rCAL) defined as (PD+rRH); (5) combined defect height (CDH), measured as the distance from the coronal to the apical margins of the noncarious cervical lesion; (6) percentage of combined defect coverage (CDC), calculated as ([preoperative CDH – postoperative CDH]/preoperative CLH) x 100 for all groups.

A calibration exercise was performed to determine acceptable intra-examiner reproducibility. The calibration was achieved by examination of 15 defects in five participants twice in a period of 72 h. Calibration was accepted if clinical measurements taken at baseline and at 72 h were similar to 0.5 mm at the 90% level^2^.

### Plaque sample collection

Sampling was performed at baseline (before the restorative-surgical treatments), and 6 and 12 months after surgery. Prior to sampling, supragingival plaque was gently removed from the gingival margin of the tooth using sterilized cotton rolls. The subgingival plaque samples were collected from the mid-buccal aspect of the gingival sulcus. Two paper points were inserted into the gingival sulcus and left in the area for 20 s^8^. They were then placed into sterile tubes containing 200 µl 1x Tris-acetate-EDTA (TAE) buffer in sterilized screw-capped cryotubes and stored at -80°C prior to DNA extraction of the samples.

### Microbiological analysis

#### Sample preparation and bacterial culture

As positive controls**,** *Pg* ATCC#33227 and *Pi* ATCC#2561 strains were cultured in Fastidious Anaerobe Agar (Merck, Darmstadt, Germany) supplemented with 10% sheep blood agar, Vit K (1µg/ml) and hemin (5 µg/ml) in an automatic anaerobic chamber (Electrotek, Devon, United Kingdom) with an atmosphere of 80% N_2_, 10% H_2_ and 10% CO_2_ at 37°C for 2–7 days. One loopful of a colony of each cultured strain was suspended in 1 ml of sterile distilled water and used for genomic DNA extraction after measuring the amount of DNA quantified by NanoDrop (ThermoFisher Scientific, Helsinki, Finland). These were used as controls.

#### DNA extraction of the sample

The subgingival samples, suspended in 200 µl 1x TAE buffer, were homogenized by vigorous mixing on a vortex. After homogenization, the genomic DNA from all of the samples was extracted using the QIAamp DNA mini Kit (cat no. 51306, Qiagen, Hilden, Germany), working with a spin colon technique and DNA purification kit (GE Health Care Bio-Science Corp., Piscataway, NJ, USA) in accordance with the manufacturer’s instructions. In order to quantify the bacterial DNA of the samples, the template control DNAs were designed according to the chosen primers and probes and purchased from the Primer Design company as adjusted in the concentration of 10^9^ copies/µl. For *Pg*, a 16S ribosomal RNA gene with the accession number AF414809; for *Pi,* a 16S ribosomal RNA gene with the accession number L16468 and the template DNA of the *Td* strain JCM 8153, with the accession number AB621358, were used as controls in the PCR and qPCR assays.

#### PCR amplification

PCR amplification was carried out in a reaction volume of 20 µl consisting of 7 µl template DNA and 13 µl reaction mixture containing 0.75 µl of each of the primers, 10 µl 2X SYBR master mix (cat no:801-520, Lot no: QP116G25001, Roche, Basel, Switzerland),1 µl Rox dye and 0.5 μl RNAse/DNAse-free water. PCR cycling was carried out in a thermal cycler (ThermoFisher Scientific, Helsinki, Finland).

## Quantification of specific bacterial species by quantitative real-time PCR

In order to quantify populations of specific bacteria in the samples, quantitative real-time PCR was performed as described below, using bacterial species-specific primers: *Pg* forward, 5’-GTAGATGACTGATGGTGA-3’; *Pg* reverse, 5’-TTATGGCACTTAAGCCGA-3’; *Pg* probe, 5’-FAM-AGAAGCCCCGAAGGGAAGA-TAMRA-3’; *Pi* forward, 5’-TTTGTTGGGGAGTAAAGCGGG-3’; *Pi* reverse, 5’-TCAACATCTCTGTATCTGCGT-3’; *Pi* probe, 5’-FAM-CGGTCTGTTAAGCGTGTTGTG-TAMRA -3’; *Td* forward, 5’-GAATGTGCTCATTTACATAAAGGT-3’; *Td* reverse, 5’-GATACCCATCGTTGCCTTGGT-3’; and *Td* probe, 5’-FAM-ATGGGCCCGCGTCCCATTAGCT-TAMRA -3’. In addition, a basic local alignment search was used to find any regions of local similarity to check the specificity of the primers, and no similarities were found. Quantitative real-time PCR amplification protocols for each bacterium were tested to confirm the protocol for the optimal performance in alignments. The protocol was then adjusted as follows for all primers: initial denaturation at 95°C for 10 min, followed by 45 PCR cycles at 95°C for 15 s for denaturation, 60°C for 60 s for primer annealing, and 72°C for 30 s for extension. The reactions were performed using Applied Biosystems SimpliAmp qPCR (Applied Biosystems, Foster City, CA, USA). The dynamic range of quantification of the PCR analysis was determined by serial dilution of plasmid generated standards for each of the chosen bacteria in the range of 10^9^–10^2^ copies/ml.

## Restorative procedures

Professional oral hygiene procedures were performed in the initial therapy in each participant, including dental scaling, polishing and occlusal adjustment, when necessary, at least 2 weeks prior to the restorative treatment. Before the restorative procedures were carried out, the NCCLs were randomly assigned using sealed-coded opaque envelopes containing the type of restorative materials. In the NCR group, cavities were filled with a nanofilled-composite (FiltekTM Supreme Plus-3M ESPE, St. Paul, MN, USA). A two-step etch-and-rinse adhesive (Adper Single Bond Plus SB, 3M ESPE, St. Paul, MN, USA) was applied to the NCCLs, and light cured for a minimum of 20 s. The NCCLs were restored with FiltekTM Supreme using a layering technique. Each layer was light-cured for 20 s. In the RMGI group, cavities were filled with Fuji Ionomer Type II LC (GC Corporation, Tokyo, Japan). Firstly, GC Dentin Conditioner was applied to the NCCLs and light cured for 20 s. Encapsulated Fuji II LC was mixed according to the manufacturer’s instructions, placed into the NCCLs and then light cured again for 20 s. In the giomer group, cavities were filled with Beautifil (Shofu Inc., Kyoto Japan). A two-step self-etching procedure, consisting of self-etching primer and fluoride-releasing bonding agent (FL-Bond II, Shofu Inc., Kyoto, Japan), was used for the NCCLs and light cured for 20 s. Beautifil, which is supplied in syringe form, was flowed into the NCCLs and then light cured for 20 s. After polymerization of the restorative materials, finishing was carried out using aluminum oxide disks of decreasing abrasiveness (Sof-Lex XT, 3M ESPE, St. Paul, MN, USA).

## Surgical procedures

Two weeks after the restorative appointment, the participants underwent surgical procedures, all of which were performed by the same expert periodontist (S.C.I.). Following local anaesthesia, all recessions in each participant were treated with modification of the CAF technique^35^. The flap design is an envelope type without vertically releasing incisions. A split-full-split thickness flap was elevated to expose at least 3 mm of the marginal bone apical to the dehiscence area. The restoration margin was then established using a diamond bur. The exposed root surface apical to the restoration was planed with curettes, and a CTG was obtained with a single incision technique^10^. Grafts were positioned to cover the exposed roots and then sutured to interdental papillae using 5-0 resorbable coated polyglactin sutures (Dogsan Surgical Sutures, Trabzon, Turkey). The flaps were coronally positioned, completely covering the combined defects. Vertical double-crossed sutures^37^ were used to stabilize the flap. No periodontal dressing was used.

## Statistical analysis

Statistical analyses were carried out using statistical software (PASW Statistics 18.0; SPSS, Inc., Chicago, IL, USA). Descriptive data [counts of the bacterial DNA copies (copies/µl) and clinical data] were reported as the mean ± standard error of the mean ( ), and counts of the bacterial DNA copies were transformed to logarithmic (base 10) values. A two-factor repeated-measures analysis of variance (RM-ANOVA) was used to evaluate the counts of the bacterial DNA copies for treatment methods and time. Comparison of the frequency detection of each periodontopathogenic bacteria between the groups was made using chi-square analysis. A two-way ANOVA with PD as the dependent variable and counts of the bacterial DNA copies as the independent variable was performed, and Pearson’s correlation coefficient was used to evaluate the relationships between counts of the bacterial DNA copies and rCAL, CDH and CDC. For all analyses, the level of significance was p=0.05.

## Results

As subgingival plaque samples could not be obtained from all patients during a 12-month observation period, a final microbiological evaluation of 17 patients (9 men and 8 women; mean age 42±6.8 years) with a total of 51 Miller class I recessions was carried out. Nine maxillary incisors, eleven maxillary canines, seventeen maxillary premolars, three mandibular incisors, five mandibular canines, six mandibular premolars were treated and plaque samples were obtained from those teeth.

Regarding BOP and PI, none of the treatment groups showed any statistically significant changes in the number of positive sites between baseline and the 12-month observation period (p>0.05). However, all groups presented statistically significant reductions in the CDH values (p<0.05). The percentage of CDC were 71.18±23.16% for the NCR+CTG group; 71.33±22.33% for the RMGI+CTG group; and 64.23±20.33% for the giomer+CTG group at 12 months postoperatively. There was no significant difference in terms of both CDH and CDC values in any of the groups at the 12-month follow-up. The clinical parameters are presented in Table 1.

**Table 1:** Clinical parameters of the treatment groups in the study follow-up periods

	Baseline				6 Months				12 Months			
	**NCR+ CTG**	**RMGI+ CTG**	**Giomer+ CTG**	**p**	**NCR+ CTG**	**RMGI+ CTG**	**Giomer+ CTG**	**p**	**NCR+ CTG**	**RMGI+ CTG**	**Giomer+ CTG**	**p**
PD	1.176 ± 0.39	1.05 ± 0.24	1.176 ± 0.52	0.126	1.29 ± 0.46	1.176 ± 0.39	1.21 ± 0.33	0.44	1.32 ± 0.71	1.23 ± 0.43	1.29 ± 0.46	0.489
rCAL	12.64 ± 0.96	12.38 ± 0.74	12.47 ± 0.92	0.305	10.89 ± 0.7	10.55 ± 0.6	10.83 ± 0.75	0.567	10.97 ± 0.92	10.7 ± 0.66	10.76 ± 0.58	0.578
CDH	3.97 ± 1.09	3.76 ± 0.81	3.94 ± 1.22	0.257	1.12 ± 0.89	1.06 ± 1.15	1.13 ± 1.42	0.32	1.15 ± 1.52	1.12 ± 2.12	1.18 ± 1.83	0.45

p<0.05 considered statistically significant for intergroup comparisons, repeated-measures analysis of variance test.

PD=Probing Depth; rCAL=relative Clinical Attachment Level; CDH=Combined Defect Height

**Figure 1:**
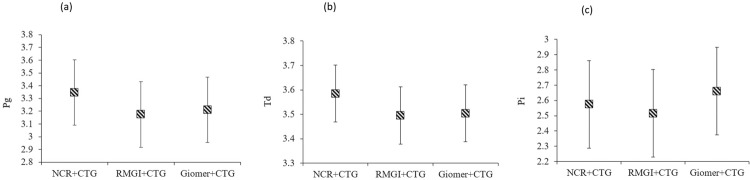
Comparisons of the counts of DNA copies of Pg, Td and Pi (copies/µl) in the treatment groups regardless of time, (a), *Porphyromonas gingivalis*, (b), *Treponema denticola*, (c), and *Prevotella intermedia*, respectively. p<0.05 considered statistically significant, repeated-measures analysis of variance test

**Figure 2:**
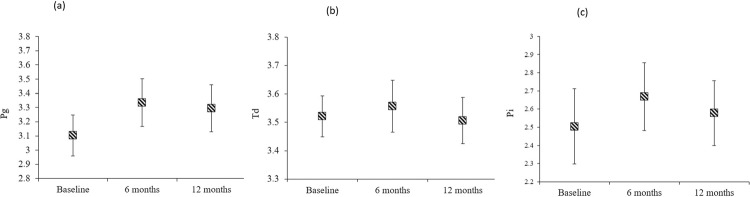
Comparisons of the counts of DNA copies of Pg, Td and Pi (copies/µl) at baseline and at 6 and 12 months postoperatively, irrespective of treatment groups, (a), *Porphyromonas gingivalis*, (b), *Treponema denticola*, (c), and *Prevotella intermedia*, respectively. p<0.05 considered statistically significant, repeated-measures analysis of variance test

**Figure 3:**
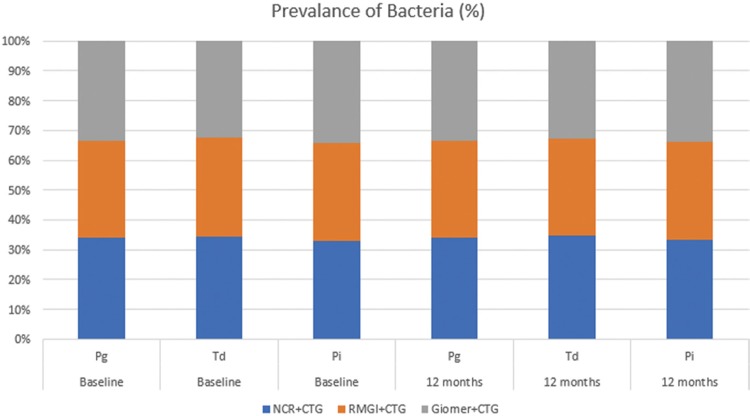
The frequency detection of Pg, Td and Pi at baseline and at 12 months postoperatively in the treatment groups. p<0.05 considered statistically significant, chi-square test

No statistically significant difference in the amount of DNA copies of *Pg*, *Td* and *Pi* was observed in any of the groups at any time points (p>0.05). In addition, there were no statistically significant differences in the counts of the bacterial DNA copies at baseline and at 6 and 12 months postoperatively, regardless of the treatment group (p>0.05; Figures 1 and 2). A reduction was observed in the detection frequency of *Td* over time, although this was not statistically significant, while the frequency of *Pg* and *Pi* was slightly higher at 12 months postoperatively compared with baseline for all groups (Figure 3).

The two-way ANOVA did not show any significant relationship between the amount of DNA copies of all bacteria and the changes in PD values (12 months after surgery and baseline) (PD<1 mm and PD≥1 mm) (Table 2).

**Table 2:** Change in counts of DNA copies of Pg, Td and Pi (copies/µl) between treatment groups with the changes of probing depth between 12 months postoperatively and baseline

	PD*<1 mm				PD*≥1 mm			
$\left(\bar{X}\pm \;S_{\bar{X}}\right)$					$\left(\bar{X}\pm \;S_{\bar{X}}\right)$			
	NCR+CTG	RMGI+CTG	Giomer+CTG	p	NCR+CTG	RMGI+CTG	Giomer+CTG	p
Pgᶧ	-0.322 ± 0.22	-0.189 ± 0.23	-0.35 ± 0.13	0.447	0.371 ± 0.21	0.139 ± 0.16	0.269 ± 0.14	0.15
Tdᶧ	0.047 ± 0.18	-0.198 ± 0.12	0.135 ± 0.09	0.052	0.078 ± 0.2	-0.166 ± 0.12	0.086 ± 0.09	0.27
Piᶧ	-0.372 ± 0.51	-0.23 ± 0.21	0.154 ± 0.2	0.297	-0.345 ± 0.34	0.174 ± 0.25	0.479 ± 0.35	0.891

* changes between 12 months and baseline in probing depth values; ᶧ changes between 12 months and baseline in counts of bacterial DNA copies; p<0.05, two-way ANOVA test

Pg=*Porphyromonas gingivalis*; Td=*Treponema denticola*; Pi=*Prevotella intermedia*

The association between changes (12 months after surgery and baseline) in counts of the bacterial DNA copies and clinical measurements (rCAL, CDH and CDC %) using Pearson’s correlation coefficient is shown in Table 3. The change in counts of *Pg’s* DNA copies was positively correlated with rCAL values at baseline in the NCR+CTG group (r=0.507, p<0.05), the RMGI+CTG group (r=0.482, p<0.05) and the giomer+CTG group (r=0.527, p<0.05). Regardless of treatment methods, positive correlations between the amount of DNA copies of *Pg* and *Pi* and rCAL and CDH values at baseline were observed at 12 months postoperatively (p<0.05).

**Table 3:** Correlations between the changes in counts of DNA copies of Pg, Td and Pi (copies/µl) and the rCAL, CDH and CDC (%) values at baseline and 12 months postoperatively

	rCAL baseline	rCAL 12 months	CDH baseline	CDH 12 months	CDC (%) 12 months
Pgᶧ					
r	0.427	0.047	0.403	0.064	0.016
p	0.000*	0.743	0.003*	0.654	0.913
Tdᶧ					
r	0.163	0.283	0.106	0.059	0.117
p	0.254	0.044*	0.461	0.681	0.413
Piᶧ					
r	0.429	0.182	0.324	0.081	0.008
p	0.002*	0.201	0.021*	0.574	0.954

* p<0.05, statistically significant correlation between Pg, Td, Pi and periodontal measurements; ᶧ changes between 12 months and baseline in counts of bacterial DNA copies;

Pg=*Porphyromonas gingivalis*; Td=*Treponema denticola*; Pi=*Prevotella intermedia*;

rCAL: relative clinical attachment level; CDH: combined defect height; CDC: combined defect coverage

## Discussion

In this study, the influence of different subgingival restorations on periodontal health was evaluated via combined restorative-periodontal treatment. The results indicate that, irrespective of the type of restorative materials, the subgingival restorations did not produce significant changes in clinical and microbiological examinations 12 months after surgery. Previous studies have reported that subgingival placement of restorative materials was more susceptible to the initation of periodontal disease by plaque accumulation and release of toxic products^5^ ^,^ ^17^ ^,^ ^29^. However, studies with a similar study design to ours showed that well-finished subgingival restorations were not associated with periodontal inflammation in combined restorative-periodontal treatment^14^ ^,^ ^21^ ^,^ ^22^ ^,^ ^24^ ^-^ ^26^.

Evaluation of differences in the frequency and quantity of the pathogens is critical in identifying the relationship between periodontopathogenic bacteria and periodontal disease. Previous studies have indicated that an increased amount of DNA copies of *Pg* and *Td* tended to be related to worsening periodontal health status^4^ ^,^ ^15^. During the 12-month observation period of our study, subgingival restorations performed with NCR, RMGI and giomer did not significantly affect the amount of DNA copies of *Pg*, *Td* and *Pi* in subgingival plaques. These findings are in accordance with those of previous studies^23^ ^,^ ^28^. Santamaria, et al.^23^ (2013) indicated that the presence of restorations in the subgingival region could not interfere with the subgingival microbiata. These authors showed that the target periodontopathogenic bacteria colonization levels were similar between a group that had been restored using RMGI and a non-restored group during the observation periods^23^. Another previous study that compared different restorative materials reported that composite resin showed some negative effects on the composition of subgingival microbiata compared with RMGI, and also that the decrease in periodontal pathogens was more evident in the RMGI group than in the composite resin group at 6 months after surgery^28^. In this study, all groups showed similar levels of bacterial colonization during the observation periods. This difference can be explained by the fact that a different type of composite material (microfilled composite resin) was used to restore the NCCLs in the study by Santos, et al.^28^ (2007). However, in our study, a nanocomposite material was used. Furthermore, the microbiological analysis method could be another reason for the disagreement between the study by Santos and our study. Counts of bacterial species were determined in samples using the checkerboard DNA-DNA hybridization technique in the study by Santos, et al.^28^ (2007). The concentration of the target bacteria in the RMGI group was lower than in the other groups in this study, with no statistically significant differences (p>0.05). This is in accordance with the results of an *in vitro* study conducted by Tarasingh, et al.^32^ (2015), and could be explained by the fact that RMGIs can inhibit the growth of some bacterial species, due to their initial low pH. In contrast with previous studies, the similarity between the results of both RMGI and composite resin in our study was due to the use of a nanocomposite material. Flausino, et al.^6^ (2014) asserted that the incorporation of nanoparticles in composite resin improved surface topography with less biofilm formation in their study. In addition, giomer, which has the properties of fluoride release and fluoride recharge potential, was associated with results that are similar to the other restorative materials in this study.

The trigger for the initiation of periodontal diseases is the presence of complex microbial biofilms. A complex consisting of *Pg, Tannerella forsythia* and *Td*, which is called “red complex”, plays important roles in the pathogenesis of periodontal disease^33^ and is highly related to clinical parameters, such as periodontal pocket depth and bleeding on probing^3^. It has also been found that the second group of bacterial species, known as the “orange complex” and including *Pi*, is also associated with clinical parameters of disease. Both complex microorganisms are generally found together, and evidence shows that colonization by the red complex species is preceded by colonization and proliferation of the orange complex^30^. In our study, no significant relationship was found between the amount of DNA copies of all bacteria and PD values in the treatment groups at any of the study periods (p>0.05). However, positive correlations between rCAL values at baseline and the change in counts of *Pg’* DNA copies between the baseline and 12 months after surgery were found for all groups (p<0.05). Similarly, several studies have reported that red complex species are highly correlated with CAL values^13^. Another parameter that correlated with counts of the bacterial DNA copies in this study was CDH values; significant positive correlations between rCAL and CDH values at baseline and both counts of DNA copies of *Pg* and *Pi* were observed at 12 months postoperatively, regardless of treatment (p<0.05). This can be explained by the fact that the higher CDH values, the more bacteria counts are found in subgingival biofilms.

In our study design, absence of a control group (CTG alone) can be considered a limitation. However, previous studies have demonstrated that the surgical procedures alone could not suffice to reduce dentin hypersensitivity and to provide better aesthetic results^21^ ^,^ ^22^ ^,^ ^24^ ^,^ ^26^ ^,^ ^36^. Moreover, well-finished subgingival restorations have not been reported to trigger development of periodontal inflammation in combined restorative-periodontal treatment^14^ ^,^ ^21^ ^-^ ^28^. Therefore, this study was hypothesized to reveal the most satisfactory type of restorative material via microbiological evaluation in the combined periodontal/restorative treatment.

## Conclusion

Within the limitations of this study, it was shown that subgingival placement of restorative materials did not negatively affect the subgingival microflora during the 12-month period after performing combined restorative-periodontal treatment. In addition, the study indicated that NCR, RMGI and giomer showed similar effects on periodontopathogenic bacteria in the treatment of gingival recessions that are associated with NCCLs.
